# Case Report: Psychosis in an adolescent with sickle cell disease

**DOI:** 10.1186/1753-2000-1-6

**Published:** 2007-07-17

**Authors:** Muideen Owolabi Bakare

**Affiliations:** 1 Federal Neuro-Psychiatric Hospital, New Haven Enugu, Enugu State, Nigeria

## Abstract

Anxiety and depression are well documented complications of adjustment in sickle cell disease (SCD), but psychosis as a direct complication of or adjustment in SCD is uncommon. This article reports a case of psychosis in an adolescent with SCD. It advocates for further study on the relationship between psychosis and brain tissue silent-infarcts in these patients and the urge for alertness on the part of health care professionals regarding a holistic approach to the management of these children and adolescents with SCD.

## Case presentation

### Reasons for evaluation

O.J, a seventeen year old male adolescent presented at the psychiatric outpatient facility of Federal Psychiatric Hospital, Calabar, Nigeria with a second episode of mental illness. He is a known sickle cell disease (SCD) patient with Hemoglobin genotype Hb.SS from South-south region of Nigeria. He was single and of Christian faith. He had just completed his high school education. He was brought by the father and an elder sister to the psychiatric out-patient facility on account of being talkative, verbal and physical aggression, poor sleep, accusing house help of witchcraft and refusal to eat his meals, believing they had been poisoned.

### History of psychiatric and general medical illness

He was apparently well until 4 weeks prior to presentation when he was noticed to be talkative and often talked out of the theme of discussion. He was easily provoked and agitated. He had physically fought the house-help on account of "discovering" her to be a witch and shouting "blood of Jesus". He had seen so many small children who were alien to the family members in their apartment, this O.J attributed to mean other members of the witchcraft society. He also saw other members of the family as being small in size in his perception while he was growing taller in size in comparison to them. He had not been sleeping adequately most part of the night. When not asleep he would be found talking to himself, praying excessively and reading the Bible. He complained that people around him knew what he was thinking without telling them. He admitted to hearing his thoughts being spoken aloud. He refused to eat the meals prepared at home, believing that the meals were poisoned and that he might be inflicted with witchcraft on eating. There were no associated depressive symptoms but he had expressed suicidal ideation in the past usually during periods of bone pain crises, however there was no definitive suicidal plan. The first episode of mental illness occurred at age fifteen and was characterized by poor sleep, visual hallucination, persecutory belief and easy irritability. It lasted only three weeks following treatment in a private hospital of which details could not be ascertained because of poor record keeping.

He was diagnosed with SCD for the first time at the age of two during a bone pain crisis. He had had blood transfusion thrice at ages four, nine and twelve years following hemolytic crises with packed cell volume (PCV) lower than eighteen percent on each occasion. He had been stable after the last blood transfusion except for intermittent episodes of bone pain crises occurring about four times a year.

### Family, development and social history

He was born to a monogamous family, third of three children of the parents. The mother died from complications of breast cancer when O.J was twelve years old. He said he missed the mother because she was most time his source of comfort during episodes of SCD crises. O.J and his two elder siblings lived with their father, a female house-help and their step-mother, whom the father married a year after O.J mother's death. O.J's siblings were well adjusted. Cordial relationships existed among the family members except for frequent frictions between O.J and the step-mother which revolved around O.J's refusals to join siblings and house-help in various house-hold chores. The father was a middle class income earner and rarely stayed around with the children because his insurance work required frequent traveling. O.J and his siblings spent most part of their time with the house-help and their step-mother. None of the patient's siblings suffered from SCD, but the father had sickle cell trait with Hemoglobin genotype (Hb.AS). There was no positive history of mental illness in the family.

Pregnancy, birth, neonatal and childhood history were uneventful except for periods of SCD crises. Developmental mile stones were within normal. He completed high school education a year prior to this episode of mental illness, made good grades and was planning further studies.

O.J was described as reserved with few friends, often kept to himself, did not like group activities and occupied his time with reading.

### Mental state evaluation

O.J was found to be frightened and agitated but well dressed and groomed. There was loosening of association in his speech and he had abnormality of thought possession. He had paranoid beliefs directed at the house-help and step-mother and experienced visual hallucinations. Orientation in place, person and time was good. Immediate and long term memory were good but short term memory was impaired and he could not concentrate. O.J had a fair judgment and insight into his clinical problem as he thought he needed some help and medications to calm his "nerves".

### Physical examination and laboratory investigations

Physical examination revealed a pale young man with a tinge of jaundice and slight dehydration. He had about four centimeter below the right costal margin hepatomegaly. Neurological Examination revealed no gross abnormality. Examinations of other systems were essentially normal. Magnetic Resonance Imaging (MRI), though recommended, could not be done on this patient because of non-availability of facility for this procedure in the geographical region where the patient was managed. Packed Cell Volume (PCV) was twenty two percent. Blood film showed reticulocytosis and one plus of malaria parasite (*Plasmodium Falciparum*). Total and differential white blood cell (WBC) counts were within normal range. Electrolytes and urea were essentially normal. Liver Function Test (LFT) did not reveal any abnormality.

### Diagnosis and treatment

The working diagnosis made on initial assessment was that of paranoid schizophrenia (F-20) and co-morbid sickle cell disease (SCD) (D-57) and malaria (B-50) based on World Health Organization (W.H.O) International Classification of Diseases, 10^th ^edition (ICD-10). Narrowing down on the diagnosis of paranoid schizophrenia without considering organic delusional (Schizophrenia-like) disorder (F 06.2) secondary to brain tissue silent-infarcts was not possible because of the limitation of in-exhaustive neurological investigation of the patient through MRI. Psychotic symptoms were treated with Risperidone 2 mg daily and he had family therapy sessions. Psychotic symptoms resolved completely after four weeks of in-patient treatment. He was encouraged to join the sickle cell club in his environment to improve his social activities and to share his experience and "pains" with other SCD patients and their parents. He was referred to a Hematologist and advised on regular hematinics and prophylactic anti-malaria intake. He was maintained on Riperidone 2 mg daily which he took for another four weeks before it was tailed off following his discharge to the psychiatric outpatient facility. Liaison service had been developed with the Hematologist in form of periodic feed back report on O.J and he had been completely stable and without psychotic symptoms in four months of follow-up.

## Discussion

Factors associated with the second episode of mental illness in O.J were identified as adjustment to coping with SCD crises, pre-morbid schizoid trait, and loss of mother through death to complications of breast cancer at the age of twelve.

Sickle cell disease (SCD) is found prevalent in black race, Arabians and the Caribbean [1]. It is a hereditary and chronic medical condition that encompasses sickle cell anemia (SCA) (Hb. SS), sickle cell hemoglobin C disease (Hb. SC) and sickle cell B thalassaemia (SB. Thal.) and the condition is characterized by red blood cell sickling patho-physiology that results in anemia, chronic organ damage, acute episodes of pain crises, infection, splenic sequestration, acute chest syndrome, stroke among others [1]. SCA (Hb. SS) is the most common of SCD and carries the most debilitating prognosis [1]. SCD occurs in about two percent of Nigerian population [2].

Adjustment difficulties have been documented among children and adolescents with chronic medical conditions like SCD [3-5]. Anxiety and depression are well documented complications of adjustment in SCD [6-9]. However, psychosis as a direct complication of SCD or of adjustment in SCD among children and adolescents is infrequently reported. Health care professionals need to be alerted on the possibility of psychosis complicating SCD among affected children and adolescents.

Considering the diagnostic limitation imposed by the in-exhaustive neurological investigation of this adolescent through MRI, the co-morbidity of psychosis and SCD in this adolescent would be discussed in light of the three possible dimensions:

### Two completely independent disease conditions co-existing

It is possible that the psychotic symptoms and SCD in this patient co-existed completely independent of each other.

### Organic psychosis complicating brain tissue silent-infarcts

Brain tissue silent infarcts are not uncommon among SCD patients [10]. Statius van Eps et al [11] reported transient psychosis in association with cerebral infarction in a thirty five year old patient with Sickle cell Hemoglobin C disease (Hb. SC). That psychosis in this patient was complicating brain tissue silent infarcts cannot be ruled out in view of the acute and transient nature of the two psychotic episodes experienced by this adolescent. Future investigation of brain tissue silent infarcts and psychosis among SCD patients is desirable because of the red blood cell sickling patho-physiologic process of the condition. Isolating brain tissue silent-infarcts as etiological factor of psychosis in SCD patients would obviously pose further treatment challenge in tackling the co-morbidity.

### Psychosocial adjustment to SCD and other life events precipitating psychosis in an adolescent with ongoing schizophrenia

Stressful life events had been associated with the onset of schizophrenia in individuals predisposed [12]. This adolescent, aside facing the psychosocial problem of adjusting to SCD lost his mother whom was his confidant to complications of breast cancer when he was twelve year old. Therefore, another explanation of the co-morbidity in this adolescent might be that adjustment to SCD and other life stressors precipitated psychotic episodes in the course of ongoing schizophrenia.

### Differential diagnosis and treatment response in this adolescent

This adolescent had experienced two episodes of acute and transient psychosis. The first episode at age fifteen lasted only three weeks and completely resolved as evidenced by the patient ability to re-focus on his academics and completing his high school education with good grades. This present episode which resolved following short duration of anti-psychotic medication had also been acute and transient, though the duration being longer than the first episode. The acute and transient nature of psychotic symptoms in this adolescent would point to a fleeting precipitating factor.

## Conclusion

Putting into consideration the three possible dimensions of co-morbidity in this adolescent each of which pose its own treatment challenges, there is need for increased awareness and collaboration using the models of consultation and liaison services among psychiatrists and other health care professionals in managing children and adolescents with SCD.

Treating psychotic symptoms in this group of patients should be with caution regarding the choice of anti-psychotics. Anti-psychotic like Clozapine that had been documented to cause agranulocytosis should be avoided in these patients that are more predisposed to bacteria infections and aplastic anemia crises.
